# The Influence of Climate and Other Agents on the Human Constitution, with Reference to the Cause and Prevention of Disease among Seamen; with Observations on Fever in General, and an Account of the Epidemic Fever of Jamaica

**Published:** 1844-07

**Authors:** 


### Art. XIX.

*The Influence of Climate and other Agents on the Human Constitution, with reference to the Cause and Prevention of Disease among Seamen; with Observations on Fever in general, and an Account of the Epidemic Fever of Jamaica.* By Robert Armstrong, m.d. f.r.s., Deputy-Inspector of Hospitals and Fleets, &c.—*London*, 1843. 8vo, pp. 208.

This work owes its origin to a circular order, " directing the principal medical officers of the naval hospitals at Portsmouth and Plymouth to deliver, during the summer months, a course of lectures to the medical officers serving in the ships-of-war in port and in the hospitals," which induced the author, in the expectation of being called upon to undertake the duties of lecturer at one of the naval hospitals, to draw up the observations now published, by way of preparation. They were originally written fifteen years ago, and are now published, with additional illustrations, from a belief that they may be useful to young medical officers entering the service, particularly to those ordered to the West Indies.

The work is highly creditable to the author, and well adapted for the great object kept in view in its composition,—" to create habits of thinking among the junior medical officers, to induce them to store their minds with useful information, to take advantage of the opportunities afforded them, of promoting the interests of science, and to apply to purposes of practical utility, the theoretical knowledge they had acquired in the medical schools."

A considerable part of the work is devoted to an examination of the effects of external agents, and more particularly changes of climate, occupation, and habits upon the health; and of the power possessed by the human constitution of adapting itself to such changes. Such a review contains, necessarily, in a work written for the purpose originally contemplated, much that is somewhat trite; but the illustration of the general principles adduced, and their application to the varied duties, in many varying circumstances, of a naval surgeon, are highly interesting and important.

There is much truth in the following observations:

" As one of the most important duties of the surgeon of a ship-of-war consists in the adoption of such means as are best calculated to preserve the health of the seamen intrusted to his care, it becomes an object of the utmost importance, that the means he recommends to his non-professional superior are founded on correct principles, and deduced from a knowledge of the exciting causes of disease. Seasoning to the climate, malaria, &c., are often used as certain cabalistical terms to explain everything, without any very precise ideas being attached to their nature, or mode of operation. The former term is generally understood to imply the power of resisting heat, as the result of habit, without any reference to those Physiological changes in the constitution which, when fully established, constitute that power of accommodation. When fever makes its appearance in a ship in harbour, malaria is the cause assigned, especially if a little mud, or a few patches of brushwood, are found on the shore, perhaps some miles distant. The seaman, on whom these influences are supposed to produce injurious effects seems to be regarded as a passive agent ready to receive impressions, whilst but little importance is attached to the various conditions under which he is placed, and the modifications produced in the physiological state of the functions of life." (p. viii.)

To the examination of those conditions and modifications, Dr. Armstrong devotes himself in such a manner as to impress his readers with the importance of looking for the obvious, predisposing, or exciting causes of disease, to which seamen are especially exposed, rather than rest satisfied with causes which are uncertain or fanciful. Such an examination leads him to important practical observations on the habits, diet, and clothing of seamen, on the fumigation and ventilation of ships, and on the means best adapted for escaping the exciting causes of disease, or avoiding the production of that susceptibility engendered by sudden changes of temperature and habits, which, in his belief, more surely occasions fever than any palludal poison. A very simple, and, as it appears to us, efficient method of maintaining a free circulation of air through the holds of ships, is suggested, on the same principles as Dr. Reid's, and having this advantage over the windsails in common use, that it requires no attention, and is attended with no inconvenience. This ventilation Dr. Armstrong proposes to effect by supporting combustion in the common fireplace, by air brought through pipes leading from the bottom of the holds, or storerooms, to the ashpit. "By this method of ventilation, every time the fire is lighted for cooking the provisions, a rapid circulation of air through the holds would be kept up by means of this simple apparatus, which requires no attention, is perfectly safe against fire, and out of the way in carrying on the duties of the ship." (p. 125.)

Dr. Armstrong examines, at considerable length, the theories of malaria or marsh miasmata, and furnishes, in addition to facts already known, many new ones, tending, as he conceives, to disprove the existence of any such agent as the cause of fever. The following are the conclusions at which he arrives:

> "All our reasonings, therefore, respecting the nature of these miasmata, are founded on an assumption; and consequently want that certainty which constitutes the only sure ground of belief. The existence of this material can only be regarded as an assertion—a species of evidence which is not admissible in the inductive sciences; and until proofs be brought forward of a very different kind from any hitherto adduced, we cannot acknowledge, without admitting contradictions and impossibilities, the existence of this vegeto-animal poison. The belief in such a material has an injurious effect, because it tends to suppress all observation and inquiry into the various causes of disease; whereas, by close observation and the correct application of well-known principles, we shall soon find that there are many powerful causes operating upon the bodies of men in warm climates, and which are fully sufficient to account for the ravages of disease, without the necessity of calling in the aid of aerial and unsubstantial phantoms." (p. 71.)

We cannot at all admit the truth of the last statement, although we believe that we are as yet in utter ignorance of the nature of the agent or agencies represented by the conventional term malaria, or marsh miasm. That there is *something* in many localities which acts as a poison on the human constitution, giving rise to fevers of an intermitting or remitting type, must, we think, be admitted by every physician of extensive experience who has practised in southern latitudes. We quarrel not about names or natures; but we maintain, boldly, that the *common* causes of disease, however "powerful," are inadequate to explain the occurrence of many fevers which we have ourselves witnessed between the tropics, as well as in Europe.

Dr. Armstrong's delineation of the symptoms of yellow fever is graphic, his description of the morbid appearances clear and precise, and his principles of treatment, in our opinion, judicious and sound. He disapproves of mercury, bleeds for excitement, and stimulates for depression; and we fear that all that has been discovered in relation to idiopathic fevers cannot lead to more than the adoption of those principles, and the experience of such observers as Dr. Armstrong to judicious rules for the time and mode of their application.

Dr. Armstrong's description of the morbid appearances in this fever does not differ materially from that of others. In his experience, alterations in the structure or appearance of the liver appear to have been less frequently observed than the descriptions of M. Louis would have led us to anticipate. Those which were observed, and the changes seen in the mucous membranes of the stomach and intestines, were frequently seen in the bodies of persons who died of other diseases.

"In our numerous dissections in this hospital (Jamaica), the mucous membrane of the stomach, in those who have died of fever, phthisis, and many other diseases, presents the same appearance. I have more than once taken the prescription-ticket of a yellow fever case, with the appearances on dissection detailed, and found the description exactly apply to the stomach in phthisis—by omitting the words 'black vomit,' the description was copied verbatim." (p. 179.)

Although not disposed to assert that yellow fever never can become contagious, Dr. Armstrong believes that, under ordinary circumstances, "there are no grounds for supposing that this fever either originates from, or is propagated by, contagion." (p. 197.) He tried the effects of inoculation with the black matter vomited, on his own person, without any effect.

In conclusion, we commend the work to the attention of our young naval and military surgeons, as one calculated to be of great service to them in the discharge of their difficult and responsible duties on foreign stations.

---

